# Barriers to Comprehensive Sexual and Reproductive Care for LGBTQ+ Individuals in Primary Healthcare: A Mixed-Methods Study Protocol

**DOI:** 10.3390/healthcare14152416

**Published:** 2026-08-05

**Authors:** Agustín López-Pavón, Eloísa Fernández-Ordoñez, Cristina Guerra-Marmolejo, Noelia Rodríguez-Losada, Laura Triviño-Cabrera, Shakira Kaknani-Uttumchandani

**Affiliations:** 1 Faculty of Health Sciences, University of Malaga, 29071 Malaga, Spain; aguslopezpavon97@hotmail.com; 2 Department of Nursing & Podiatry, Faculty of Health Sciences, University of Malaga, 29071 Malaga, Spain; cguerra@uma.es (C.G.-M.); shakira@uma.es (S.K.-U.); 3 Instituto de Investigación Biomédica de Málaga y Plataforma en Nanomedicina—IBIMA Plataforma BIONAND, 29590 Malaga, Spain; noela@uma.es (N.R.-L.); laura.trivino@uma.es (L.T.-C.); 4 Department of Mathematics Education, Social Sciences Education and Experimental Sciences Education, Faculty of Education, University of Malaga, 29071 Malaga, Spain

**Keywords:** LGBTQ+, primary healthcare nursing, sexual and reproductive healthcare, health equity, mixed-methods research, institutional discrimination

## Abstract

**Background/Objectives**: Members of the LGBTQ+ community often have poorer health outcomes and less access to health services than cisgender and heterosexual populations, and many healthcare professionals remain insufficiently aware of the barriers faced by these patients. Community nurses, given their skills and competencies, can help patients overcome barriers and achieve greater inclusion. The aim of this project is to identify the barriers and facilitators detected by the primary care nursing team of the Málaga-Valle del Guadalhorce Health District (MVGHD) when providing holistic care to LGBTQ+ individuals. **Methods**: A sequential explanatory mixed-methods design (DEXPLIS) will be employed to robustly combine a quantitative baseline of professional attitudes with an in-depth qualitative exploration of institutional discourses. Phase I consists of a pilot cross-sectional study with cluster allocation of questionnaire versions in which primary healthcare nursing staff in the MVGHD will complete the validated Sexuality Attitudes and Beliefs Survey (SABS) with one group completing a version framed around LGBTQ+ patients and the other around the general population. Phase II consists of a qualitative descriptive study based on discourse analysis (DA) using in-depth semi-structured interviews, analyzed with ATLAS.ti 23. **Conclusions**: This study protocol is expected to provide an empirical framework to identify potential institutional barriers and attitudinal biases in nursing care, highlighting the potential need for targeted training programmes in sexual and reproductive health. The resulting protocol aims to contribute to health equity by offering practical resources to optimize ongoing clinical education in primary care settings.

## 1. Introduction

Under Spanish legislation, Organic Law 10/2022 [1] regulates labour rights and obligations regarding sexual freedom, raising awareness and the defence of physical and moral integrity in the workplace. Among the competencies required in primary healthcare is that of responding to different patterns of human sexuality [2], promoting its assessment and the development of instruments for appropriate intervention and health education for different stages of life.

According to the World Health Organization [3], gender is a social factor that, together with sexual orientation, is associated with healthcare inequalities. Indeed, the WHO considers it essential to incorporate the gender perspective into healthcare in order to improve inclusion and empowerment policies for the entire population. The WHO defines health inequalities as conditions influenced by social, political and economic context and resulting from an unequal distribution of resources among people from different countries and cultures, affecting people’s standard of living, life expectancy, risk of illness and access to treatment and care.

The term LGBTQ+ refers to a group of people whose sexual orientation differs from that of heteronormativity, in terms of sex and/or gender [4], i.e., the multiple dimensions of lesbian, gay, bisexual, transgender and queer (or questioning) populations. Lesbian and gay people are women and men, respectively, who have sexual and emotional relationships with their peers, while bisexuals are those who may be physically and sexually attracted to another person regardless of their gender [5]. A transgender individual is someone who experiences an incongruence between the sex assigned at birth and their gender identity. The term non-binary encompasses the entire population that does not conform to the classical binary standards of gender (woman/man).

The term heteronormativity (or cisheteronormativity) refers to two assumptions: on the one hand, that everyone has a sexual orientation towards their opposite sex/gender; and on the other, that everyone conforms to and identifies with the sex they were assigned at birth [6,7]. In accordance with these premises, any sexual orientation or gender identity that deviates from these norms is often socially positioned as inferior; it is perceived as a threat and must be internalized.

Compared to the cisheteronormative population, the LGBTQ+ community has poorer health outcomes, with higher rates of anxiety, depressive disorders, suicide attempts, substance abuse, eating disorders, chronic diseases such as colon and gynecological cancers, and obesity and sexually transmitted diseases, due to late diagnosis, among other biopsychosocial impacts [8]. Another relevant factor is that of the medical and other complications that may arise from sex-change interventions.

At different stages of their lives, members of the queer community have specific vital needs which, in many cases, are not met [9]. Furthermore, they may experience insecurity and discomfort when receiving healthcare, which provokes a reluctance to engage with the health service [8]. The main reason for this unease is that in many instances the system, too, assumes gender heteronormativity and routinely imposes it.

To a large extent, the sexual and reproductive healthcare inequalities experienced by the LGBTQ+ community arise from discrimination based on gender identity and sexual orientation [10,11,12]. This discrimination is especially apparent in the actions of healthcare personnel, among whom nurses are the largest group and the most influential in this respect. Therefore, it is essential to identify and overcome such barriers to healthcare access and to propose inclusive alternatives.

The Spanish healthcare system is currently facing significant economic and social challenges that directly impact the health of the population. In primary care, appropriate nursing attention underpins patients’ relations with the system and fosters lasting confidence [13]. At this level, preventive medicine is delivered via health education, which should be provided within all social contexts and be free of discrimination [14,15,16].

Primary care nursing is usually the first point of reference for healthcare services [13], including those concerning sexual and reproductive health. In this respect, comprehensive but focused training for nursing staff is needed to ensure that sexual health issues are considered and treated in accordance with the specific needs and idiosyncrasies of the patient. However, studies have highlighted a lack of essential knowledge and professional experience among nurses regarding the sexual and reproductive needs of the LGBTQ+ community [17,18,19,20], partly due to inadequate training in this area.

The provision of holistic, integrated and personalized healthcare for LGBTQ+ patients is hampered by a lack of human and material resources, clinical practice guides and continuing education. In many cases, therefore, the health system environment fails to support the reproductive and sexual care of this population and does not structurally address the barriers they face [21]. Furthermore, many primary care nurses lack confidence and/or experience difficulty in addressing this population in order to provide them with essential health services, probably due to the non-development of appropriate skills and to limited knowledge about the sexual issues involved.

The cultural and religious influence on the sexual and reproductive care provided to LGBTQ+ patients both arises from and feeds back into the culture of addressing sex as a taboo subject in society, an attitude from which nurses are not exempt [22]. Specifically, members of the queer community describe having been subjected to discriminatory, prejudiced or stereotyped behaviours due to their sexual orientation or gender nonconformity. Another area about which many nurses are inadequately informed is that of fertility or reproductive options for this community, a matter of great importance to the individuals concerned [23].

In clinical manuals, homosexuality and transsexuality have historically been conceptualized as pathologies, a label that has led to unequal treatment and reinforced discrimination and stigmatization. The inclusion of these terms in medical classifications has not only legitimized their consideration as disorders but has also influenced social perceptions and the creation of structural barriers to these individuals’ recognition and rights.

In the 1973 edition of the Diagnostic and Statistical Manual of Mental Disorders (DSM) [24], homosexuality was no longer considered a disease, but it remained present under the category of ego-dystonic homosexuality until 1986, perpetuating the idea that this was a condition requiring clinical intervention. It was not until 1992 that the WHO definitively removed homosexuality from the International Classification of Diseases (ICD) [25], a milestone in depathologisation.

By contrast, transsexuality has undergone multiple changes in terminological and diagnostic criteria, which have sustained its pathologisation to this day. In the 1980 DSM-III [26], transsexuality was defined as a disorder, underlining its association with mental illness. Although the classifications have evolved, the concept remains present in the manuals, and in ICD-11 [27] it is reformulated under the term “Gender Incongruence.” While this represents progress, it maintains the idea that such identities require a medical diagnosis.

The persistence of these classifications has had direct consequences on the ways in which LGBTQ+ patients are perceived and treated, which has affected their access to rights, health services and legal recognition [28]. In response, calls have been made to question the effects of this pathologisation and to promote approaches that respect diversity, avoiding the imposition of medical categories that perpetuate inequalities.

The nurse Madeleine Leininger [19,29] has discussed the fundamental impact of culture on healthcare, observing that the beliefs of the nursing team undoubtedly influence the provision or otherwise of holistic and culturally competent care. From an anthropological perspective, these dynamics can be further understood through Mark Nichter’s framework of “Idioms of Distress,” which highlights how culturally standardized communication formats and structural stressors shape how vulnerable groups express suffering and engage with health services. In this context, healthcare barriers and heteronormative practices do not act in isolation; rather, they form a syndemic framework where structural discrimination, clinical training deficits, and social stigma interact to multiply health inequities in the LGBTQ+ population. Furthermore, an intersectional approach is required to acknowledge that these barriers are significantly modulated by age. Sexual communication, perceived barriers, and reproductive healthcare needs differ substantially across the lifespan. Older LGBTQ+ individuals often experience a double burden of ageist stereotypes and cisheteronormativity, which deeply conditions both the attitudes of health professionals and the patients’ actual access to care [30]. Therefore, raising awareness of the queer experience through an intersectional, syndemic lens would facilitate a better approach to sexual and reproductive care.

In the absence of such advances, the present reality often consists of minimal, unsafe healthcare and services, a lack of confidence among medical professionals and rising levels of harmful sexual behaviour and sexually transmitted diseases in the LGBTQ+ population [31]. The root of such problems lies in the heteronormative, patriarchal and hegemonic perspective too often adopted by nurses in their approach to and treatment of this community, thereby generating a climate of distrust and unease [17,18,20]. In general, models of heterosexual activity are still assumed and applied according to “traditional” criteria without addressing the specific needs and peculiarities of other populations, for example regarding contraception methods or hormone replacement therapies, which may be associated with unwanted events and malpractice.

From the evidence currently available, we conclude that there remains a significant gap in scientific knowledge regarding the precise mechanisms through which primary care nursing professionals deliver sexual and reproductive care to the LGBTQ+ population. While general healthcare discrimination is widely reported, there is a distinct lack of protocols designed to capture how local professional discourses, heteronormative communication frameworks (“idioms of distress”), and intersectional age dynamics operate synchronously as invisible barriers within community nursing consultations. This study addresses this specific knowledge gap by mapping both explicit self-reported attitudinal barriers and underlying symbolic resistances within professional practices.

## 2. Rationale

Calls are widespread within the LGBTQ+ population, of all ages, for nurses to provide up-to-date evidence-based sexual education, the absence of which is the main barrier to their accessing appropriate healthcare services [32,33]. This demand creates an opportunity for nursing personnel to raise awareness and actively engage with this target population, fostering their participation in the healthcare system by addressing their sexual and reproductive needs.

In view of these considerations, the present research project is aimed at exploring and addressing the major challenges facing nurses in the administration of basic care and meeting specific needs related to sexuality and human reproduction in the queer community. This process is of essential practical value in terms of clinical care since the identification of specific barriers and needs would facilitate training in this field and enhance professional practice, directly benefiting the health of these patients [34].

Since 2011, the Nursing Degree course offered at the Faculty of Health Sciences of the University of Malaga has included Gender and Sexual Health as a compulsory subject within the Basic Training syllabus (6 ECTS credits) with the following main objective: “*To acquire basic knowledge regarding gender and human sexuality, and to reflect on one’s own attitudes and those of others, to help develop a critical, open and tolerant outlook in this respect*” [35]. Another subject that has been taught since the same year is that of Transcultural Care. This also forms part of the Basic Training syllabus and is eligible for 6 ECTS credits. The main goals of this teaching are to “*equip students with the knowledge, skills, abilities and attitudes necessary to achieve the specific cognitive, procedural and attitudinal competencies required to deliver comprehensive, high-quality care to culturally diverse people, families and communities, from a transversal and multidisciplinary standpoint*” [36].

Despite the potential benefit of this university teaching, it remains essential to address the root cause of the present situation in the healthcare system. The scientific and social relevance of the proposed study lies in the sample of participants targeted, namely primary care nursing staff working in the MVGHD. These professionals were selected for study because they represent a basic pillar of healthcare and because their views directly reflect the impact of prior training [36,37]. Furthermore, in the absence of sensitivity training and comprehensive information on the question, graduates entering the nursing profession will continue to reproduce the shortcomings currently experienced by the LGBTQ+ community. In our opinion, it is of the utmost importance to identify the present gaps and needs in the area of sexual and reproductive education, to demonstrate the potential health benefits of remedial action, and to offer practical resources to those responsible for ongoing clinical training.

## 3. Objectives

The overall goal of this research project is to analyze whether differences exist in primary care nursing approaches to human sexuality counselling depending on whether the care is explicitly directed toward LGBTQ+ individuals or the general population.

### 3.1. Specific Objectives and Outcome Hierarchy

- To ensure methodological clarity and consistency, the specific objectives of this sequential explanatory mixed-methods protocol are organized into primary and secondary outcomes across both phases:

#### 3.1.1. Phase I: Quantitative Component (Pilot Cross-Sectional Survey)

- Primary Outcome: To evaluate and compare self-reported attitudinal barriers and perceived comfort scores among primary healthcare nursing professionals when providing human sexuality counselling, as measured by the total and dimensional scores of the Sexuality Attitudes and Beliefs Survey (SABS).
- Secondary Outcome: To estimate the statistical relationship between these sexuality-related attitudes/beliefs and key sociodemographic and professional variables (specifically age, sex, years of experience in primary care, and previous training in sexual diversity).

#### 3.1.2. Phase II: Qualitative Component (Discourse Analysis)

- Secondary Outcome: To analyze the discourses and interpretive repertoires of nursing professionals in order to explore the symbolic barriers, latent biases, and institutional or organizational facilitators that cannot be captured by closed-ended instruments like the SABS scale.
- Secondary Outcome: To identify the key meaning structures within professional narratives that explain potential discrepancies between theoretical comfort and actual clinical practice, using these insights to explain and enrich the quantitative findings from Phase I through methodological triangulation.

## 4. Materials and Methods

### 4.1. Study Design

This study will employ a sequential explanatory mixed-methods design (DEXPLIS). This design allows a quantitative panoramic view of perceived barriers to be obtained in a first phase (Phase I), followed by an in-depth exploration of the discourses, meanings and underlying biases of nursing professionals through a qualitative approach (Phase II). The quantitative component will be reported following relevant SPIRIT guidelines (Standard Protocol Items: Recommendations for Interventional Trials) where applicable.

### 4.2. Phase I: Pilot Quantitative Study

#### 4.2.1. Study Design and Study Population

Phase I consists of a pilot cross-sectional study with cluster allocation of questionnaire versions, in which primary healthcare nursing staff in the MVGHD will complete the validated Sexuality Attitudes and Beliefs Survey (SABS).

#### 4.2.2. Study Sample and Inclusion/Exclusion Criteria

Given the exploratory nature of this phase, the study is configured as a pilot and feasibility study. A total sample of *n* = 112 subjects is gathered via cluster allocation at the health-centre level. To justify the statistical validity of this sample size under a cluster design perspective, we consider a total of 33 health centres (clusters) with an estimated average cluster size of m approx 3.4 nurses. Assuming a conservative Intraclass Correlation Coefficient (ICC) of *p* = 0.02 for attitudinal measurements in primary care, the calculated Design Effect (Deff) is approximately 1.05 [Deff = 1 + (m − 1)*p*]. This indicates that the variance inflation due to clustering is minimal, confirming that the sample size of *n* = 112 retains sufficient statistical power for this pilot exploration while matching formal feasibility criteria.

- Inclusion criteria: Nursing professionals currently working in primary care within the MVGHD who freely accept participation as indicated by the integrated informed consent within the survey.
- Exclusion criteria: Healthcare professionals performing functions outside the scope of community nursing or working in settings other than primary care.

#### 4.2.3. Study Variables

Study variables include those related to the biopsychosocial characteristics of nursing professionals (age, sex, educational level, sexual orientation, primary care centre, employment type and years of experience in primary care) collected via an ad hoc questionnaire and the 12 items of the SABS scale (Table 1) [34].

#### 4.2.4. Procedure, Sample Size and Data Analysis

The validated Sexuality Attitudes and Beliefs Survey (SABS) [34] will be used as the measurement instrument. The sample will be divided into two groups:

- Group A: Nursing professionals who complete the SABS scale with a brief introduction making specific reference to the sexual and reproductive care of LGBTQ+ individuals.
- Group B: Nursing professionals who complete the SABS scale with a brief introduction referring to the sexual and reproductive care of the general population, without specifying sexual orientation or gender identity.

Data collection procedure:

1. Initial contact will be made with the heads of the health centres to inform them of the study objectives and obtain institutional consent. Following acceptance, cluster allocation will be performed to generate two groups (Group A and Group B). To ensure maximum methodological rigour, the cluster allocation sequence will be generated by an independent statistical researcher who is completely external to data collection and recruitment. The generation will be performed using a computerized random number generator. Allocation concealment will be maintained at the institutional level: health centres will be assigned to either Group A or Group B simultaneously, and participating nurses will be blinded to the existence of an alternative version of the survey, mitigating potential performance or social desirability biases.
2. The SABS scale will be used to evaluate attitudes and beliefs around human sexuality in nursing, with the aim of detecting obstacles in sexual counselling and recommendations.

The complete survey comprised: (1) an ad hoc section collecting sociodemographic data (age, primary care centre, nationality, sex, sexual orientation, educational level, employment type, and years of experience in primary care), and (2) 12 SABS items rated on a five-point Likert scale (1 = Strongly disagree to 5 = Strongly agree). The survey will be distributed electronically via the LimeSurvey platform, with biweekly reminders to maximize response rates.

#### 4.2.5. Statistical Analysis

Statistical analysis will be conducted using open-source software Jamovi version 2.12.0 (The jamovi project, Sydney, Australia). The analytical process will be structured as follows:

Descriptive and exploratory analysis.

Categorical (nominal) variables are expressed as absolute and relative frequencies (percentages). For ordinal variables (Likert items) and non-normally distributed quantitative variables, non-parametric measures of central tendency and dispersion use median (Mdn), interquartile range (IQR), and minimum/maximum values.

Inferential analysis and hypothesis testing.

After verifying non-compliance with normality assumptions (Shapiro–Wilk test), non-parametric tests will be applied:

- Pearson Chi-square (χ^2^) for associations between categorical variables.
- Mann–Whitney U for two-group median comparisons.
- Kruskal–Wallis H for comparisons involving more than two groups.

Robustness and variability control.

Homogeneity of variances will be assessed using Levene’s test. Where heteroscedasticity is detected, appropriate robust adjustments will be applied. A 95% confidence level and statistical significance threshold of *p* < 0.05 will be set for all analyses.

Regarding the management of missing data, the mechanism of missingness will be evaluated using Little’s Missing Completely at Random (MCAR) test; if missing data exceed 5%, Multiple Imputation by Chained Equations (MICE) will be applied. To quantify the magnitude of the differences, appropriate non-parametric effect sizes will be calculated and reported: Rank-biserial correlation for Mann–Whitney U tests and epsilon-squared for Kruskal–Wallis tests. The internal consistency of the SABS instrument within our specific study sample will be rigorously assessed by calculating both Cronbach’s alpha and McDonald’s omega coefficients. Finally, to control the type I error rate during multiple comparisons, *p*-values will be adjusted using the Benjamini–Hochberg False-Discovery Rate (FDR) procedure where appropriate.

### 4.3. Phase II: Qualitative Descriptive Study of Discourse

#### 4.3.1. Qualitative Study Design and Participants

A second qualitative phase based on discourse analysis (DA) is proposed. This approach will explore structures of thought, interpretive repertoires, symbolic resistances and attitudinal barriers in LGBTQ+ care as expressed in the professional narratives of primary care nursing staff. The central objective is to deepen understanding of the discourses, meanings and underlying biases that could not be captured by the SABS scale due to the social desirability bias detected in Phase I.

#### 4.3.2. Study Population and Sample

An intentional sample will be selected through maximum-variation purposive sampling, seeking to represent the diversity of profiles within the MVGHD, including professionals with different years of experience, educational levels, and health centres. Based on the previous literature using discourse analysis (DA) in health service research, it is estimated that between 15 and 20 in-depth interviews will be required to achieve theoretical saturation, defined as the operational point at which new data yield no additional conceptual categories or interpretive repertoires.

#### 4.3.3. Procedure

In-depth semi-structured interviews will be used as the primary data collection technique. The interviews will be conducted by members of the research team who possess specific postgraduate qualifications and formal academic experience in qualitative health research and gender/LGBTQ+ perspective studies (specifically authors EFO, SKU). A formalized, pre-tested interview protocol and semi-structured question guide will guide the process, ensuring consistency across participants while allowing space for emerging themes. The primary language utilized during the interviews will be Spanish, given the professional setting within the Andalusian Health Service; however, bilingual protocols (Spanish/English) will be available to accommodate potential diversity among participating healthcare professionals. As an optimization strategy to guarantee the authenticity of discourse and mitigate social desirability, the possibility of integrating clinical vignette elicitation will be considered, which involves presenting hypothetical scenarios to facilitate the projection of real attitudes.

#### 4.3.4. Data Analysis

Qualitative data will be analyzed via discourse analysis (DA) supported by ATLAS.ti 23 (ATLAS.ti Scientific Software Development GmbH, Berlín, Germany). The process will be structured in the following operational phases:

- Phase 1—Preparation and Transcription: Recorded interviews will be transcribed verbatim, preserving expressions, pauses and idioms. After a familiarization reading (data immersion), texts will be imported into ATLAS.ti 23 as “Primary Documents”.
- Phase 2—Open Coding (Textual Level): Inductive line-by-line coding will be performed, identifying units of meaning and assigning open codes (conceptual and in vivo). The goal is to break down discourse into its most basic components: perceived barriers, fears, beliefs and interpretive repertoires.
- Phase 3—Axial Coding and Categorisation (Conceptual Level): Resulting codes will be compared and grouped by semantic similarity to generate categories and subcategories. The ATLAS.ti 23 “Network View” (Semantic Networks) tool will be used to establish logical relationships between categories, enabling visualization of professional thought structures and potential heteronormativity biases.
- Phase 4—Integration and Triangulation (Theoretical Level): Methodological triangulation will be performed by contrasting the discourse analysis findings with the quantitative results of Phase I (SABS). Co-occurrence reports will help explain why professionals scoring high in “competence” in questionnaires manifest “terminological insecurity” or “avoidance” in their actual discourse.

Methodological Rigour and Trustworthiness: To minimize subjectivity and potential researcher bias, the qualitative phase will strictly adhere to Lincoln and Guba’s criteria for establishing trustworthiness. Credibility and confirmability will be ensured through investigator triangulation (independent open and axial coding by two separate researchers with consensus meetings) and the maintenance of a detailed reflexive research journal to track potential assumptions. Dependability will be supported by an explicit audit trail of all analytical steps within ATLAS.ti 23. Finally, member-checking will be performed by sharing verbatim summaries with a subset of participants to validate that the interpretations accurately reflect their narratives.

## 5. Discussion

This study protocol describes a sequential explanatory mixed-methods design aimed at exploring the barriers, facilitators, and competencies associated with the provision of sexual and reproductive healthcare for LGBTQ+ individuals within primary healthcare settings.

Previous research has highlighted the existence of healthcare inequalities affecting LGBTQ+ populations, including barriers related to stigma, discrimination, insufficient professional training, and limited access to culturally competent care [10,20,38,39]. Although nurses play a central role in promoting health equity and delivering person-centred care, several studies have reported gaps in knowledge, confidence, and preparedness when addressing the specific sexual and reproductive health needs of LGBTQ+ individuals [17,20,32]. Among the barriers they face is the lack of training on the specific health needs of the LGBTQ+ community, a shortcoming that generates insecurity and lack of confidence in the care provided. Moreover, the stereotypes, stigmas and prejudices related to sexual and gender diversity can have an unfavourable impact on the nurse–patient relationship, making it difficult for the parties to communicate effectively and for the patients’ reproductive needs to be properly diagnosed [10,38,39].

The quantitative phase of the present study is expected to provide preliminary evidence regarding nursing professionals’ attitudes, beliefs and perceived competencies in this area. In addition, the comparison between questionnaires framed around LGBTQ+ healthcare and those referring to the general population may help identify potential differences in professional responses that could otherwise remain unnoticed.

The qualitative phase has been designed to complement and expand these findings by exploring the meanings, experiences and interpretative frameworks underlying professional practice. Through discourse analysis, the study seeks to identify potential symbolic barriers, implicit assumptions, and contextual factors that may influence the provision of sexual and reproductive healthcare [20,30,38,39]. The proposed research is expected to emphasize that creating a discrimination-free work environment and providing ongoing training for healthcare professionals would foster inclusive, high-quality care.

One of the main strengths of this protocol lies in its mixed-methods design, which enables the integration of quantitative and qualitative evidence. This approach may facilitate a more comprehensive understanding of a complex phenomenon that cannot be fully captured through survey-based methods alone. Furthermore, the use of methodological triangulation is expected to strengthen the validity and interpretability of the findings.

Another strength is the focus on primary healthcare nurses, who often represent the first point of contact within the healthcare system and therefore play a critical role in promoting inclusive and equitable care. Understanding their perceptions and experiences may contribute to the development of targeted educational initiatives and institutional strategies aimed at improving healthcare delivery for LGBTQ+ populations.

Several limitations should also be acknowledged. For example, self-reported measures are inherently susceptible to social desirability bias, particularly when addressing sensitive topics related to sexuality and gender diversity. To mitigate this limitation, the qualitative phase will explore underlying discourses and attitudes that may not emerge through structured questionnaires.

Overall, this study is expected to contribute to the growing body of evidence concerning sexual and reproductive healthcare for LGBTQ+ populations. By identifying barriers, facilitators, and training needs among primary healthcare nurses, the findings may inform future educational programmes, organizational policies, and healthcare interventions aimed at promoting equity, inclusion, and high-quality care for sexually and gender-diverse populations [10,20].

## 6. Conclusions

This study protocol outlines a necessary framework to identify and dissect institutional discrimination towards LGBTQ+ patients within the primary healthcare system. By thoroughly analyzing both the barriers and facilitators in nursing care, this research aims to expose the systemic gaps that compromise equity and quality in healthcare delivery for sexual and gender minorities. The anticipated findings are expected to underscore a critical, undeniable need: the implementation of specialized training programmes in sexual and reproductive health tailored for nursing professionals. Addressing this educational deficit is a vital step toward dismantling institutional biases and fostering culturally competent care. Furthermore, the strategic dissemination of the results—through high-impact journals, open-access repositories, and direct communication with participating primary care centres—will not only guarantee transparency but also establish a robust, empirical foundation to guide future research, healthcare policies, and intervention strategies in LGBTQ+ health equity.

## Figures and Tables

**Table 1:** Study variables.

Variable	Classification	Values	Source
Randomisation group	Dichotomous qualitative	Group A Group B	Ad hoc questionnaire
Sex	Polytomous qualitative	Female Male Prefer not to say	Ad hoc questionnaire
Age	Discrete quantitative	20–29 30–39 40–49 50–59 ≥60 years	Ad hoc questionnaire
Nationality	Nominal qualitative	Spanish Other	Ad hoc questionnaire
Educational level	Polytomous qualitative	Nursing Diploma Nursing Degree Master’s Doctorate	Ad hoc questionnaire
Sexual orientation	Polytomous qualitative	Heterosexual Homosexual Bisexual Asexual Other Prefer not to say	Ad hoc questionnaire
Primary care centre	Nominal qualitative	33 health centres in the MVGHD: Alameda–Perchel Alhaurín de la Torre Alhaurín el Grande Álora Alozaina Campanillas Capuchinos Carlinda Carranque Churriana Ciudad Jardín Coín Colonia Sta Inés-Teatinos Cruz de Humilladero Delicias El Cónsul El Palo Estación de Cártama Huelin La Luz La Roca Limonar Miraflores de los Ángeles Nueva Málaga Palma-Palmilla Portada Alta Puerta Blanca Puerto de la Torre Rincón de la Victoria San Andrés-Torcal Tiro Pichón Trinidad Jesús Cautivo Victoria	Ad hoc questionnaire
Type of employment	Nominal qualitative	Permanent Temporary Substitute	Ad hoc questionnaire
Years of experience in primary care	Ordinal qualitative	<1 year 1–5 5–10 >10 years	Ad hoc questionnaire
SABS Scale	Likert-type scale	Strongly disagree Disagree Neutral/Undecided Agree Strongly agree	SABS Instrument

## Data Availability

Data sharing is not applicable to this article as no new datasets have been generated or analyzed yet. This manuscript describes a study protocol, and data collection will commence following the formal implementation of the described methodology.

## Abbreviations

LGBTQ+: Lesbian, Gay, Bisexual, Transgender, Queer. MVGHD: Málaga-Valle del Guadalhorce Health District. WHO: World Health Organization. SABS: Sexuality Attitudes and Beliefs Survey.
